# Draft Genome Sequence of the Ionic Liquid-Tolerant Bacterium *Bacillus amyloliquefaciens* CMW1

**DOI:** 10.1128/genomeA.01051-14

**Published:** 2014-10-16

**Authors:** Atsushi Kurata, Yuu Hirose, Naomi Misawa, Kohei Hurunaka, Noriaki Kishimoto

**Affiliations:** a Department of Applied Biological Chemistry, Graduate School of Agriculture, Kindai University, Nara City, Nara, Japan; b Electronics Inspired-Interdisciplinary Research Institute, Toyohashi University of Technology, Tempaku, Toyohashi, Aichi, Japan; c Department of Environmental and Life Sciences, Toyohashi University of Technology, Tempaku, Toyohashi, Aichi, Japan

## Abstract

Here, we report the draft genome sequence of an ionic liquid-tolerant bacterium, *Bacillus amyloliquefaciens* CMW1, which is newly isolated from a Japanese fermented soybean paste. The genome sequence will allow for a characterization of the molecular mechanism of its ionic liquid tolerance.

## GENOME ANNOUNCEMENT

Ionic liquids, which are composed of a bulky asymmetric cation and a small anion, are able to dissolve many compounds (1, 2). As an effective pretreatment in the production of valuable compounds (e.g., liquid fuels and fine chemicals) derived from various biomasses (e.g., cellulose, chitin, and keratin), an ionic liquid, 1-butyl-3-methylimidazolium chloride ([BMIM]Cl), improved the digestibility of these biomasses (3–5) but is toxic to bacteria that are used in the subsequent production steps (6). Therefore, to develop an efficient production step, there exists a demand for a bacterial host that is tolerant to [BMIM]Cl.

We isolated a bacterium, *Bacillus amyloliquefaciens* CMW1 (16S rRNA gene GenBank accession no. AB983212), from a Japanese fermented soybean paste. The strain CMW1 tolerates growth in the presence of >0.6 M [BMIM]Cl, which is toxic to most bacteria. The detailed biological data of the strain will be published elsewhere. In order to elucidate the molecular mechanism of its ionic liquid tolerance, we sequenced the whole genome of *B. amyloliquefaciens* CMW1 using a MiSeq sequencer (Illumina). A total of 930,000 paired-end reads with an insert size of 650 bp, covering a total of 225 Mbp, and 460,000 mate-paired reads with an insert size of 8 kbp, covering a total of 72 Mbp, were sequenced. The reads were *de novo* assembled using Newbler version 2.8 (Roche Applied Science, Penzberg, Germany), and the gaps between the contigs were closed *in silico* using GenoFinisher and AceFileViewer (7). We obtained eight scaffolds totaling 3,908,571 bp, with an average length of 488,571 bp (largest, 1,767,611 bp; smallest, 3,049 bp), one of which was self-circularized and predicted to be a plasmid (61,368 bp). The chromosome consists of seven scaffolds with a total size of 3,847,203 bp. The calculated G+C content of the draft genome is 45.8%. All assembly data were deposited in the DDBJ/EMBL/GenBank/nucleotide sequence database. Gene prediction and functional annotation were carried out by the Microbial Genome Annotation Pipeline (MiGAP) (http://www.migap.org [8–12]). It predicted a total of 9,175 protein-coding genes, one rRNA gene, and 1,022 tRNA genes.

Only one bacterium, *Enterobacter lignolyticus* SCF1, with resistance to the toxic effect of an ionic liquid, 1-ethyl-3-methylimidazolium chloride, has been isolated to date (13). It was suggested that strain SCF1 resists the toxic effect by pumping the toxic chemicals out of the cells and taking up compatible solutes (sugars and amino acids) from the environment (14). The RAST annotation (http://blog.theseed.org/servers/ [15]) of the genome sequence of strain CMW1 revealed 102 genes associated with stress responses and 81 genes associated with membrane transport. The stress response genes include one gene associated with detoxification and 16 genes associated with osmotic stress (1 for osmoregulation and 15 for compatible solute uptake and biosynthesis). The membrane transport genes include 18 genes associated with ABC transport and 11 genes associated with energy-coupling factor (ECF) transport. We plan to detect other genes involved in the tolerance of the toxic effect of ionic liquids in the genome of *B. amyloliquefaciens* CMW1.

### Nucleotide sequence accession numbers.

The draft genome sequence of *B. amyloliquefaciens* CMW1 is available in DDBJ/EMBL/GenBank under the accession numbers DF836084 through DF836091.
